# A model of COVID-19 transmission to understand the effectiveness of the containment measures: application to data from France

**DOI:** 10.1017/S0950268820002162

**Published:** 2020-09-22

**Authors:** P. Zongo, M. Zorom, G. Mophou, R. Dorville, C. Beaumont

**Affiliations:** 1 Laboratoire L3MA, DSI et IUT, Université des Antilles, Schoelcher, Martinique; 2 Institute 2iE, B.P. 594, Ouagadougou, Burkina Faso; 3 Laboratoire LAMIA, Université des Antilles, Campus de Fouillole, 97159, Pointe à Pitre Guadeloupe (FWI) – Laboratoire MAINEGE, Université Ouaga 3S, 06 BP 10347 Ouagadougou 06, Burkina Faso; 4 INRAE, Université de Tours, UMR Biologie des oiseaux et aviculture, F-37380 Nouzilly, France

**Keywords:** Basic reproduction ratio, containment measures, COVID-19, new wave

## Abstract

The main objective of this paper is to address the following question: are the containment measures imposed by most of the world governments effective and sufficient to stop the epidemic of COVID-19 beyond the lock-down period? In this paper, we propose a mathematical model which allows us to investigate and analyse this problem. We show by means of the reproductive number, 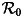 that the containment measures appear to have slowed the growth of the outbreak. Nevertheless, these measures remain only effective as long as a very large fraction of population, *p*, greater than the critical value 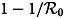 remains confined. Using French current data, we give some simulation experiments with five scenarios including: (i) the validation of model with *p* estimated to 93%, (ii) the study of the effectiveness of containment measures, (iii) the study of the effectiveness of the large-scale testing, (iv) the study of the social distancing and wearing masks measures and (v) the study taking into account the combination of the large-scale test of detection of infected individuals and the social distancing with linear progressive easing of restrictions. The latter scenario was shown to be effective at overcoming the outbreak if the transmission rate decreases to 75% and the number of tests of detection is multiplied by three. We also noticed that if the measures studied in our five scenarios are taken separately then the second wave might occur at least as far as the parameter values remain unchanged.

## Introduction

In December 2019, a disease that appeared in central China precisely in the city of Wuhan (Hubei Province) started to take its toll. On 7 January 2020, Chinese authorities admitted that the country was facing an epidemic caused by a new virus from the coronavirus family. First named ‘2019-nCOV’, this virus and disease was named COVID-19 or SARS-CoV-2 by the World Health Organization (WHO) [1]. COVID-19 disease has passed in a few weeks from a localised epidemic to a pandemic. This disease is now a public health emergency at the international level and is currently affecting more than 200 countries with more than 350 000 deaths and nearly 6 million people infected according to the WHO. It is contagious with human-to-human transmission via respiratory droplets or by touching contaminated surfaces and then touching one's face. The most common symptoms are fever, cough and difficulty breathing, but it can cause acute respiratory distress, which is often fatal.

The spread of the disease has enormous consequences for all sectors of society, endangering economics of almost all countries in the world. In the current state of knowledge, there is no preventive vaccine, biomedical means of prevention or specific therapeutic means. International, national and local control strategies are essentially based on barrier measures, social distancing, wearing masks, confinement, screening and diagnosis according to various methods and symptomatic treatment. Today in different countries, research in all its dimensions has become an absolute priority. In particular any research which can help to understand, prevent and treat COVID-19 is encouraged at the highest political level of many countries. Following this urgency, models have already been proposed in order to study the dynamics and to control the pandemic [2–9]. In this paper, we propose a new model which could help to understand the effectiveness of the containment measures adopted across countries. The model will be used to predict different scenarios of the possible resurgences of the new waves of epidemic in France.

The paper is organised as follows. The next section presents the model. The basic reproduction ratio is established in Section ‘Basic reproduction number’. Section ‘Construction of the containment rate’ is devoted to the formulation of the function which regulates the containment measures. In section ‘Model parameters’, we identified the values of model parameters. Section ‘Simulation experiments: application to data from France’ presents the simulation experiments for which five scenarios will be implemented: validation of model by comparison with the actual available data in France, testing the effectiveness of containment measures and longer-term forecasting of epidemic, study of the effectiveness of the large-scale testing, study of social distancing measures and the combined study of the large-scale testing and social distancing and/or wearing masks measures. Concluding remarks will follow in Section ‘Discussion and conclusion’.

## Model formulation

**Fig. 1.**
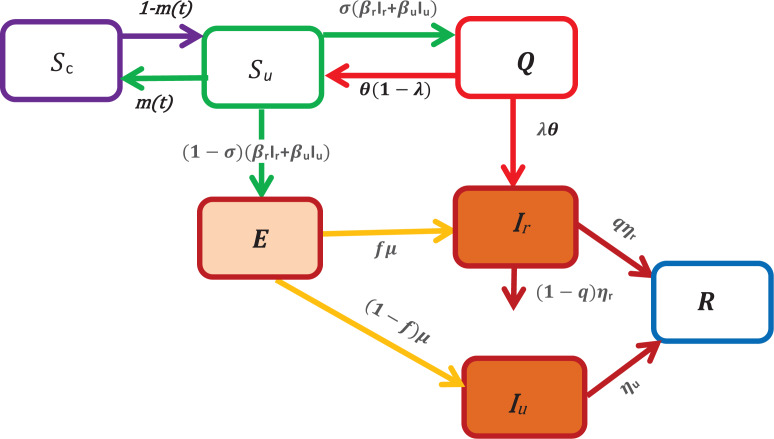
A schematic of the model for COVID-19 transmission. In this figure, *S*_u_ represents the number of unconfined susceptible, *S*_c_ denotes the number of confined susceptible, *E* depicts the number of exposed, *I*_r_ denotes the number of reported infectious, *I*_u_ represents the number of unreported infectious or silent carriers, *R* denotes the number of recovered and *Q* denotes the number of quarantined. The arrow shows the people moving between the compartments.

To model the COVID-19 transmission, we divide the human population into seven classes. Susceptible unconfined *S*_u_(*t*), susceptible confined *S*_c_(*t*), exposed *E*(*t*), reported infectious *I*_r_(*t*), unreported infectious or silent carriers, *I*_u_(*t*), quarantined *Q*(*t*), recovered *R*(*t*) at any time *t*, see in Figure 1.

In this paper, the unreported infectious individuals depict mainly the individuals with no clinical symptoms (asymptomatic or silent carriers) during their infectious period. They also include some infectious individuals with mild symptoms who thus often go unrecognised.

In what follows, we assume that these individuals don't die of the disease, while the reported infectious can die of disease at a rate (1 − *q*)*η*_r_ where 1 − *q* is the fraction of reported individuals that die, *q* is the fraction of reported individuals that recover and 1/*η*_r_ is the average length of infectious period of reported individuals (see Fig. 1).

An individual moves to the susceptible unconfined class, *S*_u_, either from the confined class at a rate 1 − *m*(*t*) or from the quarantined class at a constant rate *θ*(1 − *λ*). The fundamental parameter that we have introduced in our model to study the containment measures is the parameter *m*(*t*), it can be interpreted as the fraction of confined susceptible individuals at any time *t*. When the susceptible individuals are exposed to the virus, then the exposition provides either the reported class, *I*_r_ or the unreported class, *I*_u_. Without making any distinction about the origin of the infection, we assume that a fraction *σ* of susceptible unconfined individuals which has been in contact with an infectious individual is quarantined with contact tracing while the other fraction (1 − *σ*) who was not detected by the contact tracing move to the exposed class *E* once effectively infected or stay in compartment *S*_u_ otherwise. Then, the quantities (1 − *σ*)(*β*_r_*I*_r_ + *β*_u_*I*_u_)*S* and *σ*(*β*_r_*I*_r_ + *β*_u_*I*_u_)*S* represent the inflow of new individuals into the exposed class *E* and quarantined class *Q* respectively. The parameters *β*_r_ and *β*_u_ are the transmission rate of reported and unreported cases, respectively. We assume that reported individuals will participate in the infections with a lower rate than those unreported because they are generally isolated at the hospital or at home. However, they can transmit the infection to caregivers or their entourage. Moreover, they may have first been asymptomatic carriers contributing to the transmission of the virus. To simplify the notation, we set *β*_u_ = *β* and *β*_r_ = *ñβ*_u_ = *ñβ* where *ñ* ∈ [0, 1]. The parameter *ñ* represents the infectivity of the reported cases and for *ñ* = 1, the reported and unreported have the same level of infectivity. Among the quarantined individuals, a fraction *λ* of individuals are effectively infected and moves in the reported infectious class, *I*_r_, after an average duration of isolation, 1/*θ*, and a fraction 1 − *λ* returns to the susceptible class without being reported infectious. We assume that only a fraction *f* of the individuals of exposed class becomes reported infectious and enters to the class *I*_r_ at a rate *μ* where 1/*μ* represents the average length of the exposed period while the other fraction (1 − *f*) moves to the infectious unreported infectious class *I*_u_ at a rate *μ*.

With the above considerations, the model describing the spread of COVID-19 takes the form:1
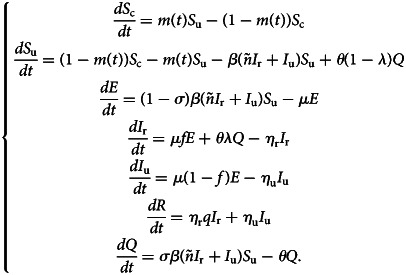


This model (1) is supplemented together with initial data *S*_c_(*τ*_0_), *S*_u_(*τ*_0_), *E*(*τ*_0_), *I*_r_(*τ*_0_), *I*_u_(*τ*_0_), *R*(*τ*_0_) and *Q*(*τ*_0_).

Let DI_r_(*t*), DI_u_(*t*), CI_r_(*t*) and CI_u_(*t*) denote the daily number of reported cases, unreported one, the cumulative number of reported cases and unreported cases respectively at any time *t*. These quantities are obtained by solving the following equations:2
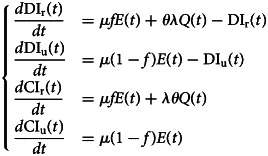
with initial conditions DI_r_(*τ*_0_), DI_u_(*τ*_0_), CI_r_(*τ*_0_) and CI_u_(*τ*_0_).

## Basic reproduction number

The fundamental key concept in epidemiology is the basic reproduction number. Commonly denoted by 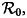 it is the expected number of secondary cases produced by a typical infective individual introduced into a completely susceptible population, in the absence of any control measure [10, 11]. Mathematically, 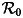 is the spectral radius of the next-generation matrix. The next-generation matrix can be obtained by construction (cf. for instance [12–14]). Using the method developed in [11], we obtain explicit formula for 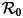 as follows:3
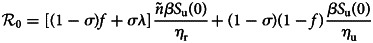
where *β*_u_ = *β* and *β*_r_ = *ñβ*. In the Appendix we give some details about the derivation of 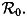


The quantity 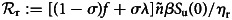 represents the average number of secondary infections produced by one reported infective individual during its infectious period, 1/*η*_r_; 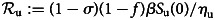 represents the average number of secondary infections produced by one unreported infective individual during its infectious period, 1/*η*_u_.

To take into account the containment measures, the large-scale testing, the social distancing and wearing masks measures, some constant parameters such as *f*, *σ*, *β* and *S*_u_(0) will be replaced in Equation (3) with the aforementioned time-dependent parameters. In this case, we can define the effective daily reproduction number, 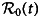 which measures the number of new infections produced by a single infected individual per day. This quantity is obtained by solving the following equation:4
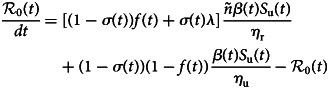
with initial condition 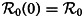 defined in Equation (3).

## Construction of the containment rate

To analyse the effectiveness of containment measures, we assume that a fraction *m*(*t*) of susceptible individuals in the population is confined at any time *t*. Furthermore, we introduce a parameter *p* which indicates the maximum percentage of the population that the government confines. This fraction should be greater than the quantity 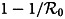 to be sure of its effectiveness [13, 15]. This parameter varies from country to country and can be set in advance for a given country. Let *τ*_0_ denotes the starting date of epidemic, *τ*_1_ represents the date at which a government decides to apply the containment measures, *τ*_2_ denotes the date at which a fraction *p* of the population is confined, *τ*_3_ stands for the date at which the government decides to exit progressively the containment measures because either the restrictions take effect or there are budget or social limitations and *τ*_*f*_ denotes the date for the end of the containment measures. Now, we divide the containment rate *m*(*t*) into four phases:

*Phase 0*: period without containment measures (from date *τ*_0_ to *τ*_1_), then *m*(*t*) = 0.

*Phase 1*: period when containment is taking place until the government reaches its maximum containment effort (from date *τ*_1_ to *τ*_2_). In this phase we assume that the function *m* increases exponentially and reach the value *p* at date *τ*_2_. It follows that *m* takes the form *m*(*t*) = 1 − exp ( − *a*(*t* − *τ*_1_)) where *a* = −ln(1 − *p*)/(*τ*_2_ − *τ*_1_).

*Phase 2*: period where the maximum effort is maintained (from date *τ*_3_ to *τ*_4_) and *m*(*t*) = *p*.

*Phase 3*: period at which the government decides to relax the containment measures (from date *τ*_3_ to *τ*_*f*_). This drop is linearly depending on the time so that the value of *m* at date *τ*_*f*_ equals to 0. Then *m* is described as follows: *m*(*t*) = *p* + *b*(*t* − *τ*_3_), where *b* = −*p*/(*τ*_*f*_ − *τ*_3_).

## Model parameters

Before to go further, let us point out that in our paper, the values of the parameters *f*, *σ*, *μ*, *θ*, *τ*_0_, *τ*_3_, *τ*_*f*_, as well as the initial values *S*_u_(*τ*_0_), *S*_c_(*τ*_0_) and *I*_r_(*τ*_0_) were chosen from expert opinions. The values of the parameters *τ*_2_, *λ*, *ñ*, *p*, *β*, *σ*, *η*_r_, *η*_u_ as well as the initial values *CI*_u_(*τ*_0_), *Q*(*τ*_0_) and *E*(*τ*_0_) were unknown. However, it is possible to identify them from specific time data. The value of the parameter *q* can be easily computed from current data. By setting *x* = (*τ*_2_, *λ*, *ñ*, *p*, *β*, *σ*, *η*_r_, *η*_u_, *I*_u_(*τ*_0_), *Q*(*τ*_0_), *E*(*τ*_0_), *τ*_2_), we estimated an optimal value of *x* that fit with the data from France by minimising the following error function:5
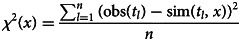
where *n* is the number of observed data, obs(*t*_*l*_) and sim(*t*_*l*_, *x*) are the observed and calculated data at time *t*_*l*_ respectively.

### Chosen values: f, σ, μ, θ, τ_0_, τ_3_, τ_*f*_, S_u_(τ_0_), S_c_(τ_0_) and I_r_(τ_0_)

*Initial conditions*: We started the simulations at the moment where in France, the number of reported cases were identified with 12 individuals, i.e. precisely on the date *τ*_0_ = 25 February. Then *I*_r_(*τ*_0_) = 12. The population of France is around 66 999 000 inhabitants [16], thus, we set *S*_c_(*τ*_0_) =66 999 000. At that date, there were no confined individuals, thus *S*_c_(*τ*_0_) = 0.

*Value of parameter f*: Recall that, (1 − *f*) stands for the fraction of exposed individuals that becomes unreported infectious and corresponds to the proportion of asymptomatic or silent carriers or mild infectious. (1 − *f*) ranges from 17.9% to 86% [1, 17–20] and some reviews therein [21]. The fractions of unreported individuals in these previous studies are derived from the number of tests performed. Therefore, the current fraction may be seriously underestimated. During the onset of COVID-19 in France, there were very little screening tests. That's why, we estimated that 1 − *f* = 0.8 (i.e. *f* = 0.2). Furthermore, we make it vary in the scenario 3 (effectiveness of the large-scale testing), as soon as the number of tests increases.

**Fig. 2.**
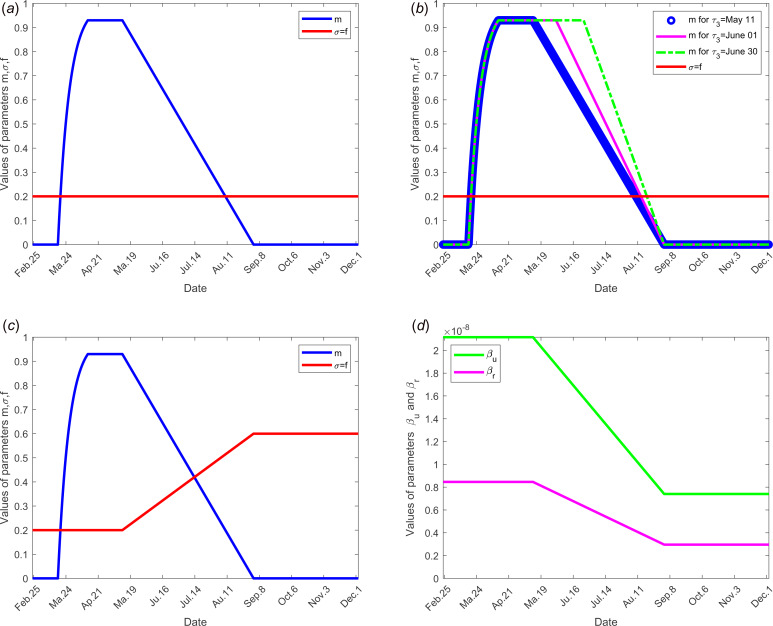
Evolution over time of parameters *m*, *σ*, *f*, *β*_r_, *β*_u_ and *τ*_3_ according to each scenario; the other parameters of the model are fixed as shown in Table 1: (a) for scenario 1, only the parameter *m* is time dependent, *f* = *σ* = 0.2, *β*_r_ = 0.846 × 10^−8^, *β*_u_ = 2.115 × 10^−8^ and *τ*_3_ = 11 May for all time. (b) For scenario 2, *f* = *σ* = 0.2, *β*_r_ = 0.846 × 10^−8^, *β*_u_ = 2.115 × 10^−8^ for all time, only the parameter *m* is time dependent for three different values of the date at which the containment measures are relaxed, *τ*_3_, more precisely when *τ*_3_ takes the values 11 May, 01 and 30 June. (c) For scenario 3, we set *τ*_3_ =May 11, *m*, *f* and *σ* evolve over time, *β*_r_ = 0.846 × 10^−8^, *β*_u_ = 2.115 × 10^−8^. (d) For scenario 4, *τ*_3_ =11 May, *f* = *σ* = 0.2,  *m* evolves as in (a), in addition, the transmission rate *β*_r_ and *β*_u_ evolve. (e) For scenario 5, *τ*_3_ = 11 May, *f*, *σ*, *m* evolve as in (c), moreover *β*_r_ and *β*_u_ evolve as in (d).

*Value of parameter σ*: The value of parameter *σ* was calibrated to 0.2 so that *σ* = *f*. In scenario 3, we assume that *f* increases in the same order as *σ* when the time evolves (see Fig. 2(c)), because when the number of tests increases, the fraction of reported cases increases and thus the fraction *σ* of susceptible unconfined individuals that is quarantined increases with contact tracing.

**Table 1.** List of parameters and their meaning and the parameter ranges for which the model was solved

Name	Meaning	Value
*p*	Maximum fraction of the susceptible that a government can confine	0.93
*f*	Fraction of Exposed that becomes reported infectious	0.4
*ñ*	Infectivity of reported individuals	0.5
*λ*	Fraction of quarantined individuals that becomes infectious	0.3
*q*	Fraction of reported individuals that becomes recovered	0.83
*σ*	Fraction of individuals which is quarantined with contact tracing	0.2
*β*_r_ = *ñβ*	Transmission rate of reported individuals	0.846 × 10^−8^
*β*_u_ = *β*	Transmission rate of unreported individuals	2.115 × 10^−8^
(1 − *q*)*η*_r_	Disease-induced death rate of reported individuals	0.016
1/*μ*	Average length of the exposed period	5
1/*η*_r_	Average length of infectious period of reported individuals	10
1/*η*_u_	Average length of infectious period of unreported individuals	4
1/*θ*	Average length of the quarantine period	14
*m*(*t*)	The fraction of confined susceptible at any time *t*	[0 − *p*], see Figure 2
*τ*_0_	Starting date of the epidemic	25 February
*τ*_1_	Starting date of the containment	17 March
*τ*_2_	Date at which a fraction *p* of the population is confined	12 April
*τ*_3_	Decision date of relaxation of the containment measures	11 May–28 June
*τ*_*f*_	End date of containment measures	01 September
Initial values	Meaning	Value
*S*_c_(*τ*_0_)	Initial confined susceptible population	0
*S*_u_(*τ*_0_)	Initial unconfined susceptible population	66 990 000
*E*(*τ*_0_)	Initial exposed population	112
*I*_r_(*τ*_0_)	Initial reported population	12
*I*_u_(*τ*_0_)	Initial unreported population	50
*R*(*τ*_0_)	Initial recovered population	0
*Q*(*τ*_0_)	Initial quarantined population	36

*Value of parameter τ*_1_: The starting date of the containment was fixed on 17 March, then *τ*_1_ = 17 March.

*Value of parameter τ*_3_: According to the announcement of French government of 13 April, a gradual deconfinement started on 11 May. So for model validation, we fixed *τ*_3_ = 11 May.

*Value of parameter τ*_*f*_: We fixed the end date of containment measures on 1 September.

*Value of parameter μ*: The mean incubation period 1/*μ* was fixed to 5 days see [1, 22, 23].

*Value of parameter θ*: We considered 14 days to isolate the quarantined individuals, therefore, 1/*θ* = 14 days.

### Estimated values: τ_2_, λ, ñ, p, β, σ, η_r_, η_u_, I_u_(τ_0_), Q(τ_0_), E(τ_0_), τ_2_ and q

By calibrating the model with the data corresponding to the cumulated reported cases for France, we identified some values of model parameters giving a good fit of the observed data obtained in [24]. The parameter values and initial conditions estimated are listed in Table 1.

*Value of parameters τ*_2_ *and p*: We estimate that the government has successfully confined 93% of the population on the date 12 April, thus, *τ*_2_ = 12 April and *p* = 0.93. This value means that 93% of the population was confined on date *τ*_2_ equals to 12 April, thus *S*_c_(*τ*_2_) = 62 300 700. In this case, 7% of the population that remained active and we set *S*_u_(*τ*_2_) = 4 689 300. Note that this number corresponds to approximately 15.78% of active population in France which was 29 700 000 according to INSEE in 2017 [25].

*Value of parameters η*_r_ *and η*_u_: By fitting with data from France, we estimate that the mean duration of infectious period for unreported individuals, 1/*η*_u_ = 4 days and for reported ones, 1/*η*_r_ = 10 days.

*Value of parameters β*_r_, *β*_u_ *and ñ*: We estimated that the infectivity of reported cases *ñ* equals to 0.40 compared to infectivity of unreported which is 1. The transmission rate *β*_u_ = *β* of unreported individuals is estimated to 2.115 × 10^−8^/day, then the transmission rate of reported individuals equals to *β*_r_ = *βñ* = 0.846 × 10^−8^/day.

*Value of parameter q*: Since 1 − *q* represents the fraction of reported individuals that dies, thus 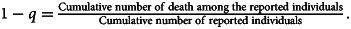


From current data (31 July), we find 1 − *q* = 30 254/186 573 = 0.1622. It follows that the disease-induced death rate of reported individuals, (1 − *q*)*η*_r_, equals to 0.1622 × 1/10 = 0.0162/day. Furthermore, the fraction of reported individuals that becomes recovered equals to *q* = 0.8378.

*Value of parameters E*(*τ*_0_), *Q*(*τ*_0_) *and I*_u_(*τ*_0_): We estimate that at date *τ*_0_, we have *Q*(*τ*_0_) = 36, *I*_u_(*τ*_0_) = 50 and *E*(*τ*_0_) = 112.

## Simulation experiments: application to data from France

### Scenario 1: validation of model with data from France

**Fig. 3.**
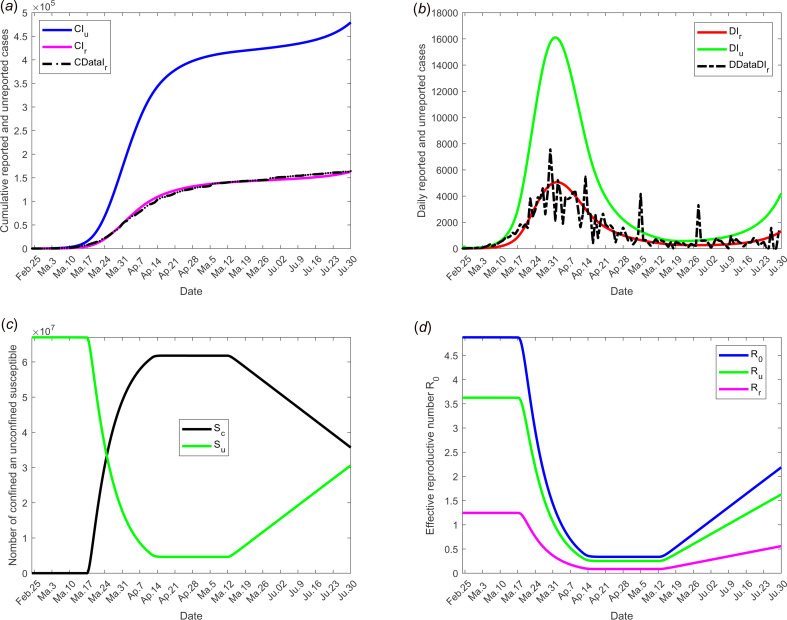
Scenario 1: Validation of model with currently data from France. The date at which the containment measures are relaxed, *τ*_3_, was fixed to 11 May; the end date of containment measures, *τ*_*f*_, was fixed to 1 September. (a) The cumulative number of reported CI_r_ and unreported CI_u_ cases simulated, and observed data CIrData. (b) The daily number of reported DI_r_ and unreported DI_u_ cases from the model and observed data DDataI_r_. (c) The confined and unconfined susceptible *S*_c_ and *S*_u_. (d) The daily reproductive number 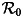. In this scenario, the values of the parameters were estimated and listed in Table 1 except for the containment function *m* which varies between 0 and 1 (see Fig. 2(a)).

We selected for model validation, the data obtained for daily reported (DRI_r_) and cumulative reported (CRI_r_) cases for France see [24]. Some constants and parameters involved in the model are listed in Table 1. The results of this scenario are illustrated in Figure 3.

### Scenario 2: effectiveness of containment measures

The objective of this scenario is to analyse if the outbreak might stop for different values of the date at which the containment measures are relaxed, namely *τ*_3_. Then, the value of the latter is assumed varying from 11 May, 1 and 30 June. The end date of containment measures *τ*_*f*_, is fixed to 1 September. The values of the parameters used are listed in Table 1 except for the containment function *m* that varies (see Fig. 2(b)).

**Fig. 4.**
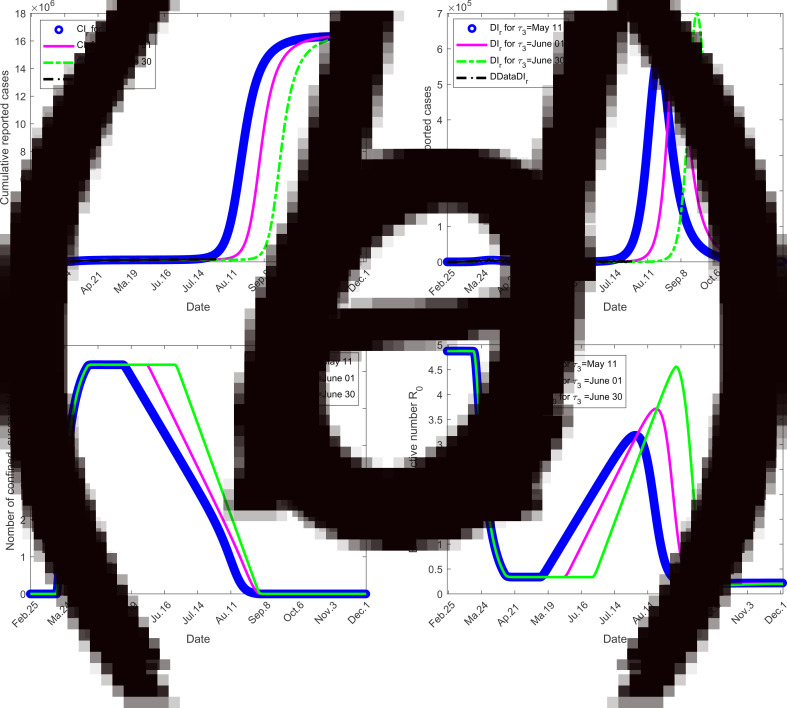
Scenario 2: Longer-term forecasting of epidemic spreading according to different values of the date at which the containment measures are relaxed, *τ*_3_ that varies between 11 May and 01 and 30 June. The end date of containment measures *τ*_*f*_ was fixed to 1 September. (a) The cumulative number of reported CI_r_ cases simulated. (b) The daily number of reported DI_r_ cases from the model. (c) The number of confined susceptible *S*_c_. (d) The daily (effective) reproductive number 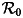. In this scenario, the values of the parameters were estimated and listed in Table 1 except for the containment function *m* which varies between 0 and 1 for different values of *τ*_3_ (see Fig. 2(b)).

The results of this scenario are illustrated in Figure 4.

### Scenario 3: effectiveness of the large-scale testing

To investigate the effectiveness of the large-scale test of detection of infected individuals, the date at which the containment measures are relaxed, *τ*_3_, is fixed to 11 May; the end date of containment measures, *τ*_*f*_, is fixed to 1 September. We assume that between the dates *τ*_3_ and *τ*_*f*_, the fraction of reported cases *f* increases linearly and reach 200% of its initial value and the fraction of susceptible individuals which is quarantined *σ* is also increased linearly to reach 200% of its initial value (see Fig. 2(c)), the initial values is estimated in scenario 1. The values of the other parameters are listed in Table 1 except for the containment function *m* that varies (see Fig. 2(b)).

**Fig. 5.**
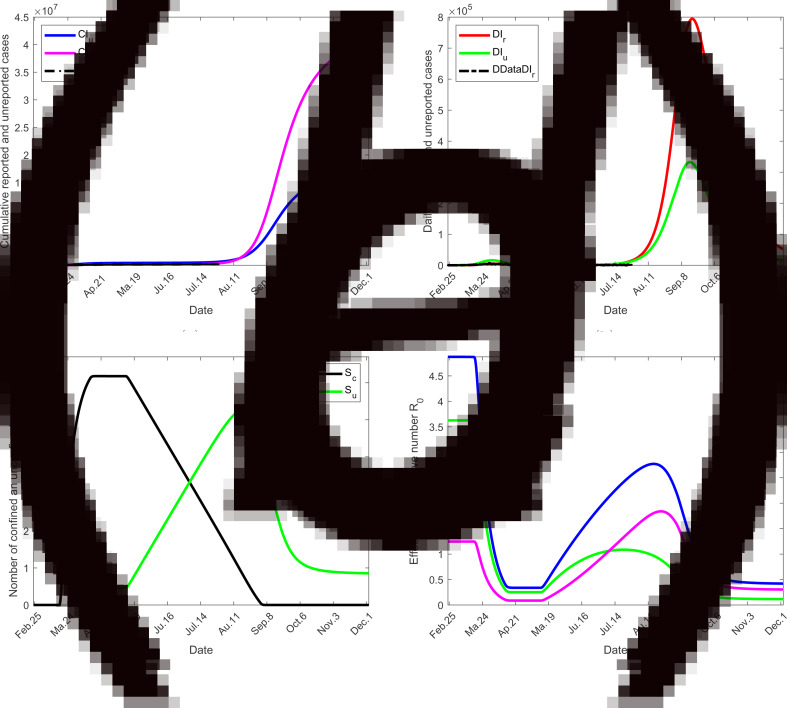
Scenario 3: Longer-term forecasting of epidemic spreading in case of large-scale tests of detection on infected individuals. *τ*_3_ was fixed to 11 May; *τ*_*f*_ was fixed to 01 September. Between the dates *τ*_3_ and *τ*_*f*_, the parameter *f* was assumed to increase linearly to reach 200% of its initial value, *σ* increased linearly to reach 200% of its initial value (see Fig. 2(c)). The values of the other parameters are listed in Table 1 except for *m* that varies (see Fig. 2(b)). (a) The cumulative number of simulated reported CI_r_ and unreported CI_u_ cases and observed data CIrData. (b) The daily number of simulated reported DI_r_ and unreported DI_u_ cases and observed data DDataI_r_. (c) The number of simulated confined and unconfined susceptible *S*_c_ and *S*_u_. (d) The daily reproductive number 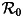.

The results of this scenario are illustrated in Figure 5.

### Scenario 4: social distancing and/or wearing masks measures

To study the social distancing and/or wearing masks measures, the date at which the containment measures are relaxed, *τ*_3_, was fixed to 11 May; the end date of containment measures, *τ*_*f*_, is fixed to 1 September. We assume that between the dates *τ*_3_, and *τ*_*f*_, the transmission rate *β* decreases linearly to reach 75% of its initial value (see Fig. 2(d)). The values of the other parameters are listed in Table 1 except for the containment function *m* that varies (see Fig. 2(b)).

**Fig. 6.**
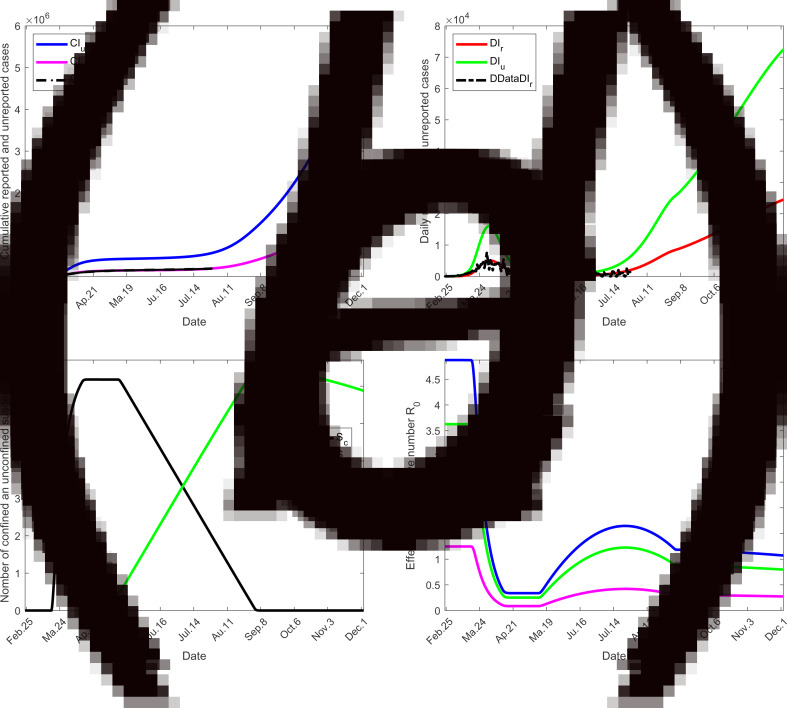
Scenario 4: Longer-term forecasting of epidemic spreading in case of the social distancing and wearing masks measures. *τ*_3_ was fixed to 11 May; *τ*_*f*_ was fixed to 01 September. Between the dates *τ*_3_ and *τ*_*f*_, the parameter *β* decreases of 75% of its initial value (see Fig. 2(d)). The values of the other parameters are listed in Table 1 except for *m* that varies (see Fig. 2(b)). (a) The cumulative number of simulated reported CI_r_ and unreported CI_u_ cases and observed data CIrData. (b) The daily number of simulated reported DI_r_ and unreported DI_u_ cases and observed data DDataI_r_. (c) The number of simulated confined and unconfined susceptible *S*_c_ and *S*_u_. (d) The daily reproductive number 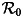.

The results of this scenario are illustrated in Figure 6.

### Scenario 5: combined effects of large-scale testing and social distancing measures

To test the combined effects of large-scale testing and social distancing social and/or wearing masks measures, we combine the conditions of scenarios 1 and 2. The date at which the containment measures are relaxed, *τ*_3_, is fixed to 11 May; the end date of containment measures, *τ*_*f*_, is fixed to 1 September. Between the dates *τ*_3_ and *τ*_*f*_, we assume that *f* increases linearly to reach 200% of its initial value, *σ* increases linearly to reach 200% of its initial value and *β* decreases of 75% of its initial value see Figure 2(d) for these different variations of parameter values.

**Fig. 7.**
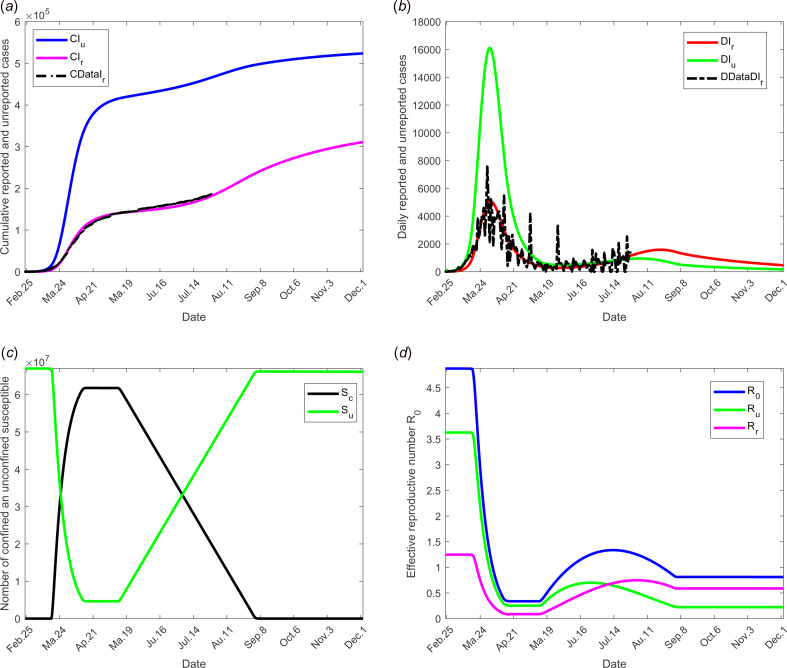
Scenario 5: Longer-term forecasting of epidemic spreading in case of both social distancing and/or wearing masks and large-scale tests. *τ*_3_ was fixed to 11 May; *τ*_*f*_ was fixed to 1 September. Between the dates *τ*_3_ and *τ*_*f*_, the parameter *f* increases linearly to reach 200% of its initial value, *σ* increases linearly to reach 200% of its initial value (see Fig. 2(c)). *β* decreases to 75% of its initial value (see Fig. 2(d)). The values of the other parameters are listed in Table 1 except for *m* that varies (see Fig. 2(b)). (a) The cumulative number of simulated reported CI_r_ and unreported CI_u_ cases and observed data CIrData. (b) The daily number of simulated reported DI_r_ and unreported DI_u_ cases and observed data DDataI_r_. (c) The number of simulated confined and unconfined susceptible *S*_c_ and *S*_u_. (d) The daily reproductive number 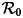.

The results of this scenario are illustrated in Figure 7.

## Discussion and conclusion

This model takes into account the measures of confinement, distinguishing between confined individuals, quarantined individuals and isolated individuals. Many values were estimated to fit the beginning of expansion of disease in France, other were inferred from expert opinions, see Section ‘Model parameters’. The proportion (1 − *f*) ranges from 17.9% to 86% [1, 17–20] and some reviews therein [21]. The lower value was observed on board of the Diamond princess, i.e. in conditions which are not representative of large-scale populations living in larger surfaces. On the other hand, in [26] and in a WHO report, it was estimated that between 80% and 86% of all infections were undocumented. Since this fraction is dependent on the number of tests performed and since during the onset of COVID-19 in France, there were fewer screening tests, we eventually chose *f* = 0.2.

As for *f*, the mean duration of infectious period is a debatable point. In [27], the authors estimate that the asymptomatic individuals had median virus persistence duration of 8.87 days (95% confidence interval 7.65–10.27). This duration varies also between asymptomatic and symptomatic individuals [28], even when mildly affected. By contrast, all severe cases were still tested positive at or beyond day 10 post-onset [29]. This longer virus persistence in severe cases as compared to milder cases has been also demonstrated by [30] but not by all authors, see some reviews therein [21]. Note also that in the literature, the estimate of the mean duration of infectious period of reported individuals is not always clear, since some authors include hospitalisation or isolation period, others do not. Results vary between 2 and 8 days [26, 31, 32]. Our model implicitly takes into account a combined effect of duration of infectivity and viral load, which results in risk of transmission. Indeed, the mean duration of infectious period estimated to be 10 days for reported individuals is coherent with the estimation in [33] based on clinical, microbiologic, epidemiologic and clinical data. Since we estimate the infectivity of the reported cases to be equal to 0.4, that of unreported cases in term of duration of infectious period, it means that reported individuals are infectious for 40% of their infectious period, i.e. 4 days. If the infectivity is interpreted in terms of viral load, it means that 40% of the viral load excreted by the infectious reported cases is infective. Such a link between duration of infectious period and infectivity (i.e. interpreted in terms of viral load) also holds in the literature except in [34] where the authors observed no difference in viral load between asymptomatic and symptomatic patients. In [35] the virus level in the asymptomatic group was significantly lower than that in the symptomatic group in the acute phase.

Figure 3 shows the adequacy of the model for predicting the evolution of number of cases in the beginning of the crisis until end of June. It also shows that as soon as confinement is reduced or stopped, the daily reproduction number 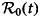 value increases again and a new wave of epidemics is to be expected as soon as its value is higher than 1. As observed in Figure 4 (for longer-term forecasting), such waves are expected to appear very shortly after reduction of confinement, once the incubation period is spent. These values will allow predicting the effectiveness of the containment measures as well as risk and the intensity of possible resurgences of the new waves of epidemic in France. Indeed, the measures of confinement have a strong impact on the value of the daily reproduction number 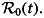 Figure 4 shows that, while it was equal to nearly 5 in the beginning of the disease, before confinement, it decreased to about 0.5 as long as confinement of most people takes place and increased to a lower value, between 2 and 2.5, that is about half its former value. But even if its value is reduced, it remains higher than 1. It is also to note that, unexpectedly, its value was lower when end of confinement was earlier. Figures 3 and 4 show that the current increase in the number of cases could be expected as soon as containment was relaxed from mid-May to September. However, the number of cases are expected to decrease if a higher proportion of infected people are detected and confined, which is currently the case. Delaying the very starting date of deconfinement to 30 June would have resulted in a later and higher wave but not as late as could be expected for a starting date to 11 May. All French people are expected to be either reported or unreported infected individuals at the end of 2020, i.e. before expected development of vaccines. However, some of those values may change with evolution of measures of prevention such as social distancing and/or wearing masks, large-scale testing, treatment of the disease.

*Social distancing and wearing masks measures* directly influence the transmission rate which is expected to dramatically decrease with the increasing tendency to wear masks. Its effect was investigated in scenario 3 where the transmission rate was assumed to decrease linearly to reach the value 75% of its initial one as shown in Figure 2(d). The results of this scenario are shown in Figure 5. The transmission rate may be dramatically decreased. But the results show that this measure alone is insufficient to eliminate the disease.

The effectiveness of the *large-scale test of detection* of infected individuals was analysed and shown in Figure 6. The fraction of reported cases, from date *τ*_3_ to *τ*_*f*_, the fraction of reported cases *f* was assumed increase linearly to reach the value 200% of its initial one and the fraction of susceptible individuals which is quarantined *σ* was also increased linearly to reach the value 200% of its initial one (see Figs 2(b) and (c)). These values may be observed by tracking all former contacts of any newly reported case, and systematically testing them. As in the former scenario dates of relaxation and end of containment measures were fixed to 11 May and 1 September respectively. Results show that this measure without further action is also insufficient to control the outbreak.

The *combined measures* of large-scale testing and social distancing and/or wearing masks measures was studied in scenario 5. The transmission rates (reported and unreported individuals) were assumed to decrease from 75% to date *τ*_2_ to *τ*_*f*_ (see Fig. 2(d)). Figure 7 shows the effectiveness of these combined measures and the potential of such a strategy. In particular, it shows that predicted data are compatible with the current situation with no evidence yet of a second wave. This result also shows that protective measures must be maintained for a long term before the hypothesis of a second wave may be discarded.

In the absence of any control measure, the basic reproduction number 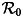 is equal to 4.8739. Most of this value is due to the weight of transmission by unreported individuals 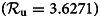 and the weight of transmission by reported cases accounts for much less 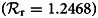. These values show that the major number of secondary infections is produced by the unreported individuals. With increasing use of appropriate tests, reported individuals will be more precisely diagnosed, thus the fraction of reported cases *f* will increase and thus the importance of 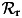 in the total value of 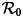. Since the infectivity of reported individuals was estimated at 0.4 compared to infectivity of unreported the increase in 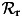 will be very small showing the importance of detecting infectious individuals; the evolution over time of the effective reproductive number 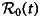 and effective weight of transmission 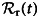 and 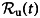 are shown in Figure 7(d). Therefore, the reported individuals will have a lower propensity to transmit the virus. Through stronger measures of prevention, the probability of contaminating other people will be lower.

With the confinement measures, the minimal percentage (critical fraction) of susceptible individuals that should be confined to eliminate the COVID-19 equals to 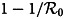 (see for instance [13, 15]). By confining more susceptible individuals, we increase the kinetics of elimination of the disease. By fitting the model with the French data, we estimated this fraction to *p* = 93%. This value belongs to the critical interval, namely 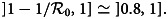


By analysing the results of simulations, we can conclude that the containment measures appear to have slowed the growth of the COVID-19 outbreak. Our model predicts that a second big wave of the epidemic may not be avoided if the situation remains unchanged and if the French government does not maintain the current efforts on large-scale tests, obligation of wearing masks inside and in some cases outside and other prophylactic measures. However, it also shows that these measures are efficient to avoid such a risk, thus preserving public health and avoiding a new confinement and all its terrible consequences, see Figures 3 and 4. While if no measures were implemented, even only one infected individual in the population would result in a new wave of infections and a new period of confinement. Some obligations will succeed in avoiding a second wave of COVID-19.

In this paper, we formulated a new model to describe the spread of COVID-19 to understand the effectiveness of the containment and quarantine measures. It is able to reproduce observed data from France and probably other countries.

## Data Availability

The datasets generated during the current study are graphical represented in the scenarios 1-5. The datasets from France used to fit the model during the current study are freely available via online public domains [25].

## Appendix

### Some details about the derivation of

In order to define  for model (1), we begin to find the disease-free equilibrium point by letting the compartments *S*_c_, *Q*, *E*, *I*_r_, *I*_u_, and *R* be zero and *S*_u_ = *S*_u_(0).

Let  denotes the inflow of new individuals into the infected classes *I*_r_, *I*_u_, *E* and *Q*

and  denotes all other flows within and out of the infected classes,

Let  and  be the Jacobian matrices of the maps  and , respectively, evaluated at the disease free equilibrium:

A straightforward computation shows that

Following [11], the matrix *FV*^−1^ is well defined, and is the next-generation matrix and  is the spectral radius of *FV*^−1^.
